# The American Dental Association

**Published:** 1882-09

**Authors:** 


AMERICAN JOURNAL

OF

DENTAL SCIENCE.

Vol. XVI. THIRD SERIES—SEPTEMBER, 1882. No. 5

### ARTICLE I.

### *American Dental Association.*

The twenty-second annual meeting of the American Dental Association was held at the Highland House, Cincinnati, August 1, 2, 3, and 4, 1882, Dr. H. A. Smith, of Cincinnati, in the chair, and Dr. George W. Cushing, of Chicago, Secretary.

Dr. A. Berry, the oldest dental practitioner in Cincinnati, delivered an address welcoming the Association to the Queen City of the West, which on account of the modesty of the President, was responded to by the First Vice-President, Dr. W. C. Barret, of Buffalo, N. Y.

Drs. Field, Buckingham, and Taft were appointed a committee to draft suitable resolutions on the death of Dr. D. Hawxhurst.

Drs. Crouse, Shepard, and Pierce were appointed as a committee to draft resolutions on the death of Dr. M. S. Dean, of Chicago.

On motion of Dr. Barret, the Secretary was instructed to send the sympathies of the Society to Dr. Marshall H. Webb, of Lancaster, Pa., who is lying dangerously ill.

#### First Day.—Afternoon.

The President, Dr. A. H. Smith, of Cincinnati, read the annual address, of which the following is an abstract:

After eulogizing the talents and social qualities of the late Dr. M. S. Dean, of Chicago, he said, in his address last year the President had called attention to the fact that etiology—the science of the causes of dental caries—had been almost wholly neglected by essayists who had contributed to the proceedings of the Association, and suggested to the section embracing etiology that a committee be appointed for the object of making a careful, systematic study of the causes which contribute to the existence of dental caries. When it was considered that much the larger proportion of their time spent in actual practice was occupied in the treatment of lesions caused by the almost universally prevalent disease, which were denominated dental caries, and that notwithstanding the subject had been extensively studied, the fact confronted them that the efficient cause of dental caries was still a matter for speculation, it was noteworthy, as well as encouraging, that the subject received so large a share of attention before the section devoted to the consideration of diseases of the teeth at the International Medical Congress, held in London last year. With the view of stimulating investigation in this direction, he would respectfully suggest that the Association offer a prize to members, of not less than $200, for the best paper based strictly upon original investigation relating to etiology of dental caries, the award to be made and announced at the annual meeting in 1884. If there was any deficiency in the treasury for that purpose, it might be met by an increase in the annual membership fee, or the whole amount may be made up by voluntary subscription of the members. Whether dentistry should be taught in special colleges or in a regular university course with other studies, was a matter that might safely be left to time, the great arbiter of all things.

Dr. Wm. H. Atkinson, of New York, Chairman of Section 3, "Dental Literature and Nomenclature," read the report of that section. It was a continuation of the same subject embodied in the reports of that section for the last three or four years. In his report he endeavored to explain a new system of language, based on the supposition that all languages were one and the same. He explained the meaning of two words, bauski and vauski, the former referring to organic esmalogy, the latter to inorganic esmalogy or biology. There were eight divisions of biology, according to the eight vowel sounds.

At the conclusion of his remarks, to which the Association had listened without understanding a word, Dr. Geo. Watt, of Xenia, O., said that the report was entirely irrelevant and foreign to the purpose of the section under which it was read, and as this was not a philological association, he hoped they would not be bored with any more such reports. So he moved the report be referred to a special committee of five to decide whether it should be published or not.

After a somewhat heated discussion by various members of the Association, Dr. George Watt arose to explain his motion. He had always been a great friend of Dr. Atkinson. Still the Doctor's judgment had been purely philological; it had not been on dental nomenclature. The society for the last four years had not been benefited by the discussion. And now came a continuation of the subject with the same animus from the same animile. There was not an original thought in the paper. Its thoughts were the vaporings of Stephen Pearl Andrews. Hence he had put his motion to appoint a committee, in order that it might be decided whether the society records should still be burdened with such useless matter. He did not care what the committee would do; whether they decided to send the essay to the Patagonians, or whether they bound it in morocco or calf—calf would be better—or distributed it for the enlightment of the people, so long as they disposed of it. Perhaps it would be better to send the paper back to its original author. However, under no circumstances could he be tortured into any feelings of disrespect against the Doctor. He was a great man, but the subject matter was entirely irrelevant to the object of the meeting. Should it be decided to put the paper on record, he would be pleased to learn every word of it by heart.

The motion was carried, and Drs. Keely, Odell, Friedrichs, Rehwinkel and Pierce were appointed as the committee.

SECOND DAY—MORNING.

The privileges of the floor were accorded to Prof. Mayr' of Springfield, Mass., who, after thanking the Association for the compliment, said: There is too much taken for granted. When I began to study the cause of dental caries I supposed that the subject had been thoroughly investigated, but judge of my surprise when I found sixty per cent. of lime salts in the carious dentine; and yet they talk of the "acid theory." It is so with other subjects as well.

Dr. C. N. Pierce, chairman of the committee appointed to investigate the merits of the paper on philology, read by Dr. W. H. Atkinson at the opening session, made the following report:

The special committee to whom was referred a paper on philology, read by Dr. W. H. Atkinson, as chairman of the section on "Dental Literature and Nomenclature," would respectfully report that they have carefully considered the subject referred to them, and are of opinion that the paper under consideration is foreign to the purpose of the section under which it was read, and that if the Association desire further communications upon general philology, that a section of that title be created; but in view of the fact that at previous meetings of this Association similar papers have been received without adverse criticism, they would suggest that this one be allowed to take the usual course, and go with other reports to the Publication Committee,

Dr. W. N. Morrisson, of St. Louis, Mo., then read a paper entitled "Some Thoughts on the Correction of Irregularity," of which the following is a synopsis:

During the whole course of his professional life he had only made one mistake in regulating teeth. In that case the patient never developed to normal size; small in statue, diminutive in physique, with large, good teeth in a small mouth, he was free to admit that she would look a little better with that part of her anatomy less prominent. But look at the thousands of cases to the other extreme. The public were clamorous for deformed mouths. The first demand was to have all teeth extracted, and have all artificial little, narrow, white teeth; second choice, about the half of their teeth extracted to improve their appearance and keep them from decaying; and thirdly to have the crowns of the remaining teeth horribly mutilated by filing and grinding.

It was a disgrace to the profession that there were so many claiming to be progressive dentists, who yielded to the demands and practiced to their requirements. A few months ago the public and a few silly snobs, through the press went into ecstasies over Patti's small mouth and beautiful teeth, while in reality it was a deformity—teeth irregular, one canine so much out of the arch that when she cast a bewiching smile, her lip sometimes caught upon it, and it was with some difficulty that she could get it down. Her profile had that sorrowful, dinged-in appearance so common at this time. Several years ago the papers had given an account of the selection of a characteristic American female head by the designer of the new silver dollar, that should accurately represent the correct type of American beauty. He did not know the lady, nor her dentist, nor did he have any information in regard to the condition of her teeth or articulation; but seeing her face upon the few dollars which had passed through his hands, he would venture the assertion that her mouth did not contain thirty-two normally formed teeth. In treatment of cases the great difficulty was to control the patients and parents. Teeth were easily moved if the force be applied in the right direction, but they are as easily returned to their old places. He therefore had decided objections to all of the complicated apparatus, and these were obviated by his system of regulating with screws and levers and rubber ligatures, which were secured to the teeth by thin annular bands or ligatures of platinum, cemented to the teeth with oxyphosphate of zinc, where they remained until the operation was entirely completed.

Dr. W. H. Atkinson regretted to see so little inclination to discuss the paper. There were a good many aphorisms in it that were orthodox and in accord with nature with few exceptions. The screw should never be used to move a tooth; a very slight force, constantly acting, is all that is necessary. There is a dearth of knowledge in regard to regulating teeth. The constant irritation of a tooth is wrong. We are now able to do in weeks what formerly required months. After we get the teeth in position, most of them will stay there without retaining plates. The idea of giving a patient a plate with screws, levers and watch keys was an abomination. The speaker then proceeded to illustrate his method, which consisted of a thin band of iridium and platinum, which extended around the anterior teeth and had its extremities cast in a plate of Reese's metal, said plate extending over the grinding surfaces of all the molars, and having depressions for the articulation of the molars of the opposite jaw. The speaker concluded by emphatically saying that this is *the* way of regulating teeth.

Dr. C. N. Pierce, of Philadelphia: That may be *the* way of regulating *some* teeth, but there are many cases in which it is no more applicable than it would be to the sole of the foot. All cases cannot be treated alike.

Dr. Shepard, of Boston: Did I understand Dr. Atkinson to say that the plate was never removed for cleansing from the beginning of the treatment to the end?

Dr. Atkinson: Yes, the plate is not removed at all during treatment.

Dr. Shepard: Any appliance that cannot be removed and cleansed by the patient is not a good one. Such plates *can* be made. One defect in Kingsley's "Oral Deformities" is that in two hundred pages on this subject there are only three lines on cleansing the regulating apparatus.

Dr. McKellops, of St. Louis, spoke of Dr. Coffin's method of employing piano wire. An impression cup is fitted to the mouth and gutta-percha, previously softened in hot water, placed therein. The cup and contents are then immersed in cold water for a few seconds, introduced into the mouth, pressed into position, allowed to remain a few moments, withdrawn and placed in cold water, to allow any part that has been disturbed on removing the cup to resume its proper shape.

A plaster model is made in the usual way and sheet wax spread over it, covering the molars. A piece of piano wire, 15 gauge, is then bent in the shape of a written letter *m*, with the initial and terminal stroke lengthened these ends are flattened, but *never heated.* The wire is immersed; in a solution of chloride of zinc and then dipped in melted tin, this coating preventing the sulphur attacking the wire. The ends of the wire are imbeded in the wax, and the whole thing vulcanized, finished up and fitted into the mouth. After the plate had been worn a few hours, the plate is to be cut through the median line with a fine saw and the parts spread apart somewhat, and make a thick plate and insert a piece of sea-tangle and depend on its expansion to move the tooth. I often have patients come to me with the first molars wholly demoralized, and in such cases I remove them, but do not find that the second molars tip, but that they come forward and fill up the space.

Dr. C. N. Pierce, Philadelphia: Economy of time and simplicity of appliances are of much importance. In regulating retreating upper incisors I take a piece of platanized gold, twenty-four gauge and about one-sixteenth of an inch wide, and make each end pointed. I then drill a small hole in the tooth to be moved and one in the first molar and spring this piece in place. It is not in the way as it lies along the lingual surfaces of the intervening teeth. If the tooth does not remain in position a retaining plate should be made. The strip of gold should have a hole drilled near one end so that it can be tied in to prevent its being swallowed. The small pits can be filled after the teeth are regulated.

Dr. I. N. Crouse, of Chicago, cites a case of contracted arch in the lower jaw that he had succeeded in expanding by means of jack-screws.

Dr. B. G. Marklein, Milwaukee (to Dr. McK.): Are these plates as good for drawing teeth in as for forcing them out?

Dr. McKellops: Certainly; I have just passed a plate around illustrating that.

Dr. Marklein: In a case where the only anchorage to be had is the second superior molars. is that sufficient for drawing back the other ten?

Dr. Darby: In such a case I would wedge the bicuspids back and then draw the incisors and canines into place.

Dr. Morrison: The original Coffin plate was one with dove-tails opposite the teeth to be moved. in which were inserted pieces of wood, the expansion of which moved the teeth. I have made quite a number of these plates and accomplished good results with them, but I offer in my paper a substitute for so many appliances that require ligatures.

SECOND DAY—AFTERNOON.

The committee on resolutions, in reference to the death of Dr. D. C. Hawxhurst, reported a paper which was ordered spread upon minutes. It recognized the capabilities of the deceased, and stated that he died when he had before him a greater sphere of usefulness. The heartfelt sympathy of the Association was tendered to the stricken wife.

Dr. A. G. Rawls, Lexington, Ky., then read a paper on "Pulpless Teeth," (which, owing to the peculiar and rapid delivery of the speaker, I was unable to report.—*Reporter.*)

Dr. Harlan, of Chicago, read a paper on the "Treatment of Alveolar Abscesses," in which he advocated the use of hydrogen peroxide and gave his mode of preparing it, saying that he preferred an etherial solution to an aqueous one, as it was more stable.

Prof. Mayr: I have met with many persons who make use of the term vis vitæ, and yet I have not heard any one define it.

Dr. Rawls: It is a mysterious force.

Dr. Mayr: It is a mere empty word I would like to know just how much vis vitæ is exerted. In regard to hydrogen peroxide, I suggested some time ago that it might be used as a bleaching agent, but the trouble is to apply it. We must first answer the question, "what colors a tooth," before we decide on a bleaching agent, There is, however, a diversity of opinion on this point. I believe it is due to hæmatine in some form. The antiseptic properties of this compound are due to the ozone liberated on decomposition of $H_2 O_2$.

Dr. L. C. Ingersoll, Keokuk, Iowa: I have written several papers on this subject and I wish to call attention to ulceration that begins at the apex and extends to the neck. I venture to say that no pulp dies spontaneously without affecting the peridental membrane. What called my attention to this lesion was the extracting of a tooth that I had failed to cure and finding about one fourth of an inch of the apex covered with calculus. The query was where did it come from? I did not have it analyzed. It is not found in connection with alveolar abscess, but with alveolar ulceration. All the ingredients found in calculus exist in the blood, and that is my reason for calling it sanguinary calculus.

Dr. Pierce: Do you find the process broken down in such cases.

Dr. Ingersoll: Yes, and that may be the source from which the calculus is derived, but I do not believe it to be the chief source, as the amount of calculus is far greater than that of the broken down process.

Dr. Pierce: I wish to endorse the views of Dr. Ingersoll in regard to the source of calculus.

Dr. Friedrichs, New Orleans (to Dr. I.): How many cases have you met with?

Dr. Ingersoll: I saw a good many cases before I knew what they really were. Since that I have had some eighteen or twenty cases.

Dr. Morgan, Nashville: Do you not find it in the so-called Riggs' disease.

Dr. Ingersoll: It is one of the most prominent characteristics of Riggs' disease.

Dr. Rawls: There are two ways by which inflammation can take place in the tissues surrounding such teeth. It is due either to some external cause that dips down from the outside, or to one that cuts off all nutritive material. The source of calculus is no doubt the one to which it is attributed. No part of the body is exempt from such deposits, but they occur only on free surfaces, and they can only occur in tissues in which an opening has been caused by inflammation. I do not consider it a result of Riggs' disease, but a concomitant of the breaking down of tissue from mercury, etc. We are in the habit of teaching our patients that we can save such teeth, that we can cause healthy tissue to grow over dead tissues and expect such live tissue to lie in contact with it when it is not possible.

Dr. Watt: We have been blundering over salivary calculus till Dr. Ingersoll gave it a name. There was a sanguinary calculus weighing thirteen ounces taken from the abdominal muscles. It is only necessary to have a nucleus and it will grow. I am unable to say what caused the calculus, but it is possible that it is due to inflammation, a portion becomes dead and a portion of the blood is dropped. As long as saliva holds free $CO_2$ in solution it holds the various salts in solution, but if the $CO_2$ is not present in sufficient quantities the salts are deposited. Whenever ammonia is present it is apt to unite with the $CO_2$ and a deposit results. The same effect is produced by alkaline mouth washes. Ammonia arises from a general breaking down of tissues, it sometimes comes from the mucous follicles, but more commonly from the breath.

Dr. Ingersoll: When we consider that the blood contains all the ingredients of calculus and that calcareous degenerations are very common in all parts of the body, it does not require a very great stretch of imagination to believe that this is sanguinary calculus. The ingredients of salivary calculus exist in the blood before they got into the saliva. The only case I saw where there was drainage was the case of one-fourth inch of the apex incrusted with calculus that I mentioned.

Dr. T. L. Buckingham, Philadelphia: It is very easy to build up a theory, but it should be founded on facts. Whenever you have a solid dead nucleus, bullets, splinters, etc., you have a deposit on them. $H_2$ $O_2$ unstable, and when it is decomposed nascent oxygen is liberated which acts directly on the tissue, it is in the form of ozone which is a molecule of three atoms. For several years I have been using a formula of Dr. C. E. Kirk, of Philadelphia. It consists of sulphate of soda and borax ground together. This is inserted into the tooth for bleaching purposes.

The borax decomposes the sulphate of soda and liberates sulphurous acid, which has quite an affinity for oxygen and thus decomposes the coloring compound; it has the advantage over chlorine of going to a greater depth. It is more permanent and simple.

Dr. W. H. Morgan, Nashville, Tenn: I think that there are no layers of periosteum in the sockets. There is no such thing as perfect success in the treatment of such cases. I think it is absurd to talk of pus secreting surfaces. They can only take from the blood what is in it, and pus does not exist in the blood.

Dr. Atkinson said he was sorry that as a body they were getting as crazy and "Atkinsonian" as himself. They were all troubled by an ignorance of the laws of terminology. What was ulceration but the breaking open of an abscess? What was suppuration but an inflammation, and inflammation an oxydation? He blessed the Lord that the ladies wore their hair long, because they retained all their inspiration. He believed in vis vitæ, and that all power came from God.

[TO BE CONTINUED].

---

